# A Single Amino Acid in the Polymerase Acidic Protein Determines the Pathogenicity of Influenza B Viruses

**DOI:** 10.1128/JVI.00259-18

**Published:** 2018-06-13

**Authors:** Joon-Yong Bae, Ilseob Lee, Jin Il Kim, Sehee Park, Kirim Yoo, Miso Park, Gayeong Kim, Mee Sook Park, Joo-Yeon Lee, Chun Kang, Kisoon Kim, Man-Seong Park

**Affiliations:** a Department of Microbiology, Institute for Viral Diseases, College of Medicine, Korea University, Seoul, Republic of Korea; b Division of Emerging Infectious Disease and Vector Research, Center for Infectious Disease Research, National Institute of Health, Korea Centers for Disease Control and Prevention, Osong, Republic of Korea; c Division of Viral Diseases, Center for Laboratory Control of Infectious Diseases, Korea Centers for Disease Control and Prevention, Osong, Republic of Korea; d Division of Viral Disease Research, Center for Infectious Disease Research, National Institute of Health, Korea Centers for Disease Control and Prevention, Osong, Republic of Korea; Icahn School of Medicine at Mount Sinai

**Keywords:** adaptation, influenza B virus, pathogenicity, polymerase

## Abstract

Influenza B virus (IBV) is one of the human respiratory viruses and one of the targets of seasonal vaccination. However, the bifurcation of two antigenically distinct lineages of IBVs makes it difficult to arrange proper medical countermeasures. Moreover, compared with pathogenicity-related molecular markers known for influenza A virus, little has been known for IBVs. To understand pathogenicity caused by IBVs, we investigated the molecular determinants of IBV pathogenicity in animal models. After serial lung-to-lung passages of Victoria lineage B/Brisbane/60/2008 (Vc_BR60) and Yamagata lineage B/Wisconsin/01/2010 (Ym_WI01) viruses in BALB/c mice, we identified the mouse-adapted Vc_BR60 (maVc_BR60) and Ym_WI01 (maYm_WI01) viruses, respectively. To find a molecular clue(s) to the increased pathogenicity of maVc_BR60 and maYm_WI01, we determined their genetic sequences. Several amino acid mutations were identified in the PB2, PB1, PA, BM2, and/or NS1 protein-coding regions, and one concurrent lysine (K)-to-arginine (R) mutation in PA residue 338 (PA K338R) was found in both maVc_BR60 and maYm_WI01 viruses. When analyzed using viruses rescued through reverse genetics, it was shown that PA K338R alone could increase the pathogenicity of both IBVs in mice and viral replication in the respiratory tracts of ferrets. In a subsequent minireplicon assay, the effect of PA K338R was highlighted by the enhancement of viral polymerase complex activity of both Vc_BR60 and Ym_WI01 viruses. These results suggest that the PA K338R mutation may be a molecular determinant of IBV pathogenicity via modulating the viral polymerase function of IBVs.

**IMPORTANCE** To investigate molecular pathogenic determinants of IBVs, which are one of the targets of seasonal influenza vaccines, we adapted both Victoria and Yamagata lineage IBVs independently in mice. The recovered mouse-adapted viruses exhibited increased virulence, and of the various mutations identified from both mouse-adapted viruses, a concurrent amino acid mutation was found in the PA protein-coding region. When analyzed using viruses rescued through reverse genetics, the PA mutation alone appeared to contribute to viral pathogenicity in mice within the compatible genetic constellation between the IBV lineages and to the replication of IBVs in ferrets. Regarding the potential mechanism of increased viral pathogenicity, it was shown that the PA mutation could upregulate the viral polymerase complex activity of both IBV lineages. These results indicate that the PA mutation could be a newly defined molecular pathogenic determinant of IBVs that substantiates our understanding of the viral pathogenicity and public health risks of IBVs.

## INTRODUCTION

Influenza virus causes seasonally recurring respiratory diseases in humans. Along with the two subtypes (H1N1 and H3N2) of influenza A virus (IAV), the two distinct antigenic lineages of influenza B viruses (IBVs), Victoria and Yamagata, increase the burden of seasonal influenza (1, 2). Based on the most recent update (as of 22 January 2018) of influenza activity by the World Health Organization (3), IBV detection rates have increased during the 2017-2018 season worldwide, and more than 89% of IBVs were found to belong to the Yamagata lineage. In contrast to IAVs, which infect a variety of animal hosts, including humans, IBVs are restricted mostly to humans (4). There have been only a limited number of studies reporting IBV infection in seals (5) and the detection of IBV-specific antibodies in a sizable portion of domestic pigs (6).

Several molecular determinants for host adaptation of IAVs have been reported. One example is an amino acid signature at polymerase basic 2 (PB2) residue 627 (7). Most avian IAVs retain glutamic acid at this residue (E627), whereas human IAVs have lysine (K627). The PB2 E627K mutation of avian IAVs has been suggested to be associated with pathogenicity in mice (8–11). Polybasic amino acids at the hemagglutinin (HA) cleavage sites of highly pathogenic avian influenza (HPAI) H5 and H7 subtype viruses may be another example of the pathogenic determinants of IAVs (12, 13). In the cases of HPAI viruses, the proteolytic cleavage of HA0 into HA1 and HA2 can be achieved by a ubiquitous protease, such as furin (14), which exists in almost all human cells. This is one of the reasons that HPAI viruses can cause systemic infection (15). Stalk truncation of neuraminidase (NA) protein has also been suggested as one of the pathogenic determinants of IAVs (16–18). It was first recognized as an evolutionary adjustment of avian IAVs from natural-reservoir waterfowls to domestic poultry species. Recently, this phenomenon was observed in the NA of H7N9 virus, and it was demonstrated that H7N9 NA stalk truncation would increase pathogenicity (19). In addition to these molecular pathogenic determinants, there have been other adaptive genetic mutations of IAVs suggested to have contributions to pathogenesis (20, 21).

Compared with that for IAVs, however, the characterization of IBV pathogenic determinants has rarely been performed. Even the mouse-adapted B/Lee/40 virus, which has been used for decades, has not been characterized in terms of the genetic determinant(s) of its mouse pathogenicity. Moreover, the characterization of mouse-adapted IAVs usually seeks to find clues related to the virulence of a zoonotic IAV that will ultimately affect humans, but the zoonosis of IBVs has rarely been reported. Hence, identifying a common denominator for the enhanced pathogenicity of IBVs, such as the E627K PB2 mutation of avian IAVs, may be of great importance because such defined IBV pathogenic determinants might be useful for the interpretation of IBV virulence and host range. To our knowledge, only a few reports have characterized mouse-adapted IBVs and suggested some amino acid mutations responsible for the enhanced pathogenicity in mice (22–27) and increased transmissibility in ferrets (28).

Here, we report a single PA mutation as a new pathogenic determinant of IBVs. We identified this concurrent PA mutation after the independent adaptation of Victoria and Yamagata IBV strains in mice. It was then confirmed, using viruses rescued through reverse genetics, that the PA mutation is responsible for the increased viral pathogenicity by modulating viral polymerase complex activity. Similar effects of the PA mutation were also demonstrated in a ferret model. Finally, we discuss the significance and potential applicability of the PA mutation in terms of pathogenesis and the genetic constellation of IBVs.

## RESULTS

### Adaptation and lethality of IBV strains in mice.

**FIG 1:**
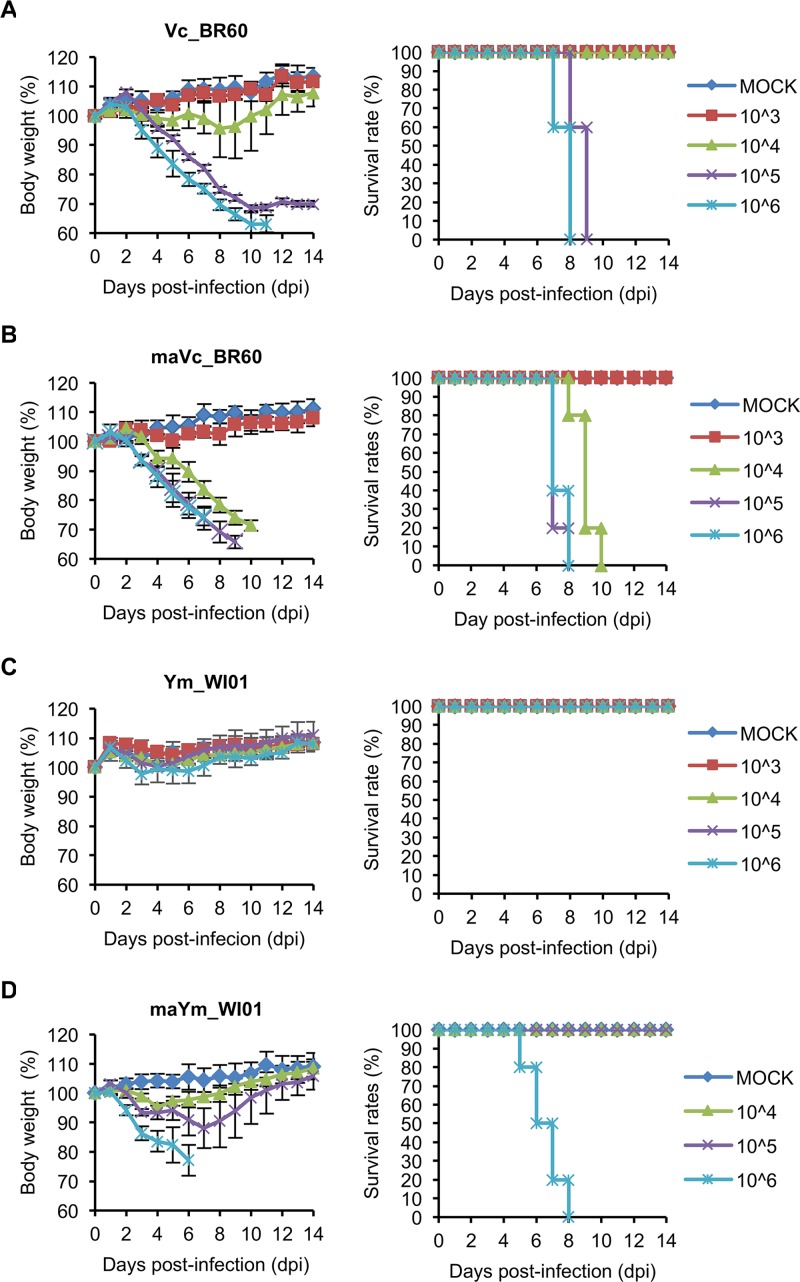
Pathogenicity of Vc_BR60 and Ym_WI01 before and after adaptation in mice. Five mice per group were infected with 10^3^ to 10^6^ PFU of Vc_BR60 (A), maVc_BR60 (B), or Ym_WI01 (C). For maYm_WI01 (D), five BALB/c mice per group were infected with 10^4^ to 10^6^ PFU. Body weight changes and survival rates of the infected mice were monitored for 14 days. Phosphate-buffered saline (PBS) was used for mock infection. Error bars for body weight changes denote standard deviations (SD).

In the determination of 50% mouse infectious doses (MID_50_) of wild-type (WT) Vc_BR60 and Ym_WI01 viruses in BALB/c mice, both viruses exhibited similar infectivity, and the MID_50_ titers of Vc_BR60 and Ym_WI01 were determined to be 10^2.7^ and 10^2.8^ PFU, respectively (Table 1). However, the viruses exhibited quite different lethalities. When infected with 10^5^ and 10^6^ PFU of Vc_BR60, the mice were all dead at 9 and 8 days postinfection (dpi), respectively, whereas all the mice infected with Ym_WI01 (10^3^, 10^4^, 10^5^, and 10^6^ PFU) survived until 14 dpi (Fig. 1A and B). Given the body weight changes and survival rates of the infected mice, 50% mouse lethal doses (MLD_50_) could be determined only for Vc_BR60 with 10^4.5^ PFU (Table 1).

**TABLE 1:** Infectivity and lethality of the Vc_BR60 and Ym_WI01 viruses in mice

Virus	MID_50_ titer^*a*^ before adaptation	MLD_50_ titer^*b*^:	
		Before adaptation	After adaptation
Vc_BR60	10^2.7^	10^4.5^	10^3.5^
Ym_WI01	10^2.8^	>10^6^	10^5.5^

a MID_50_, 50% mouse infectious dose (PFU).

b MLD_50_, 50% mouse lethal dose (PFU).

To obtain a lethal Ym_WI01 strain in BALB/c mice, we adapted Ym_WI01 through serial lung-to-lung passage. Vc_BR60 was also passaged in the mice. After adaptation, both Vc_BR60 and Ym_Wi01 showed increased lethality to the mice. As well as the 10^5^ and 10^6^ PFU titers, even the 10^4^ PFU titer of mouse-adapted Vc_BR60 (maVc_BR60) killed all the mice (Fig. 1C). Mouse-adapted Ym_WI01 (maYm_WI01) appeared to exhibit increased lethality, and the mice infected with 10^6^ PFU of maYm_WI01 were killed starting from 5 dpi (Fig. 1D). Given the body weight changes and survival rates of the infected mice, MLD_50_ for maVc_BR60 and maYm_WI01 were determined with 10^3.5^ and 10^5.5^ PFU, respectively (Table 1). In these experiments, Vc_BR60 appeared to be more lethal to the mice than Ym_WI01 before and after adaptation (Table 1).

### Mouse lung pathology of mouse-adapted IBV strains.

**FIG 2:**
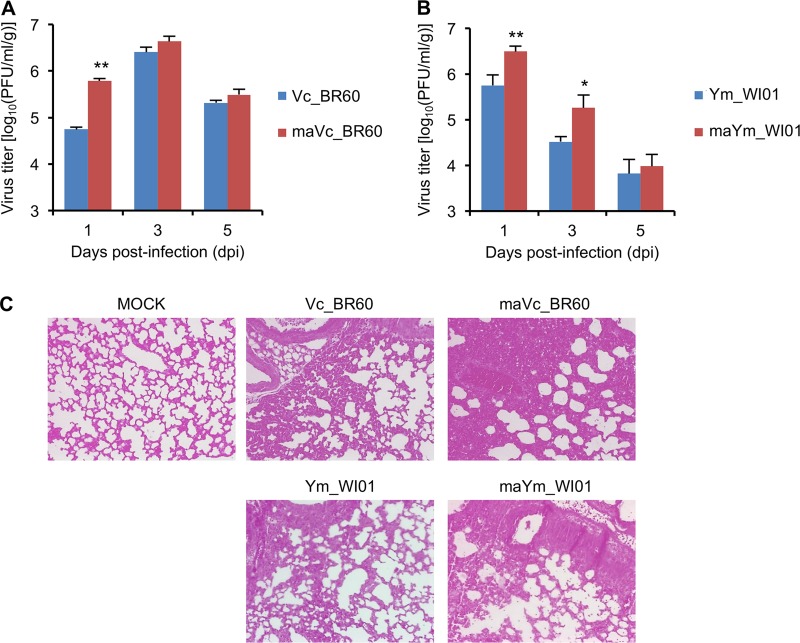
Viral replication and histopathology assessments of Vc_BR60 and Ym_WI01 in the lungs of mice. (A and B) Viral replication of Vc_BR60 (WT and mouse-adapted strains) (A) and Ym_WI01 (WT and mouse-adapted strains) (B) in the mouse lungs was determined at 1, 3, and 5 dpi. The results were averaged from the viral titers determined from three different mice. PBS was used for mock infection. Error bars denote SD. *, *P* < 0.5; **, *P* < 0.01 (compared with the titers of Vc_BR60 and Ym_WI01, respectively). (C) For lung histopathology assessment, H&E-stained slides were examined under a light microscope (magnification, ×100).

Next, we determined viral replication and histopathologic transformation in the lungs of infected mice. As expected from the MLD_50_ titers of the viruses (Table 1), Vc_BR60 exhibited higher replication titers than Ym_WI01, and the mouse-adapted viruses (maVc_BR60 and maYm_WI01) replicated in higher titers than the respective parental viruses (Fig. 2A and B). Between the two lineages of viruses, the parental and mouse-adapted Vc_BR60 viruses resulted in the highest titer at 3 dpi (Fig. 2A), whereas the Ym_WI01 counterparts started to be cleared from the lungs as early as 3 dpi (Fig. 2B). This might indirectly substantiate the virulence differences between Vc_BR60 and Ym_WI01, as represented with the MLD_50_ of the viruses (Table 1). However, given the lung histopathology of the infected mice (Fig. 2C), the severity of viral pathogenicity could hardly be distinguished between Vc_BR60 and Ym_WI01. Only the massiveness of hemorrhage and destruction of alveoli in the infected mice may indicate that maVc_BR60 and maYm_WI01 appeared to be more pathogenic than their respective parental viruses.

### Determination of genetic sequences of mouse-adapted IBV strains.

To identify the genetic basis of the increased viral pathogenicity of maVc_BR60 and maYm_WI01, we determined the genetic sequences of the viruses. Compared with the genetic sequences of the parental viruses, amino acid mutations were identified in five and three protein-coding regions of maVc_BR60 and maYm_WI01, respectively (Table 2). Specifically, maVc_BR60 appeared to harbor six amino acid mutations, in PB2 (Q78K), PB1 (A193T), PA (K338R and E550K), M2 (M21I), and NS1 (E162G), whereas three mutations were detected in maYm_WI01, in PB2 (P295T), PB1 (A541T), and PA (K338R). Interestingly, both mouse-adapted viruses appeared to have the common amino acid mutation PA K338R. The mutations in PB2, PB1, M2, and/or NS1 might collectively affect the virulence of the mouse-adapted viruses to various degrees. However, we focused on the concurrent PA K338R mutation of both the maVc_BR60 and maYm_WI01 viruses, and to investigate the pathogenic effects of this mutation, we generated mutant viruses harboring the PA K338R mutation in the genetic backbones of Vc_BR60 and Ym_WI01 (rVc_BR60_PA_:K338R and rYm_WI01_PA_:K338R, respectively) by reverse genetics (29) and used them for subsequent analyses.

**TABLE 2:** Amino acid mutations identified in the maVc_BR60 and maYm_WI01 viruses

Protein-coding region	Amino acid mutation^*a*^ in:	
	maVc_BR60	maYm_WI01
PB2	Q78K	P295T
PB1	A193T	A541T
PA	K338R, E550K	K338R
BM2	M21I	None
NS1	E162G	None

a Amino acid mutations are identified based on the genetic sequences of the respective parental Vc_BR60 and Ym_WI01 viruses.

### PA K338R mutation as a pathogenic determinant of IBVs in mice.

**FIG 3:**
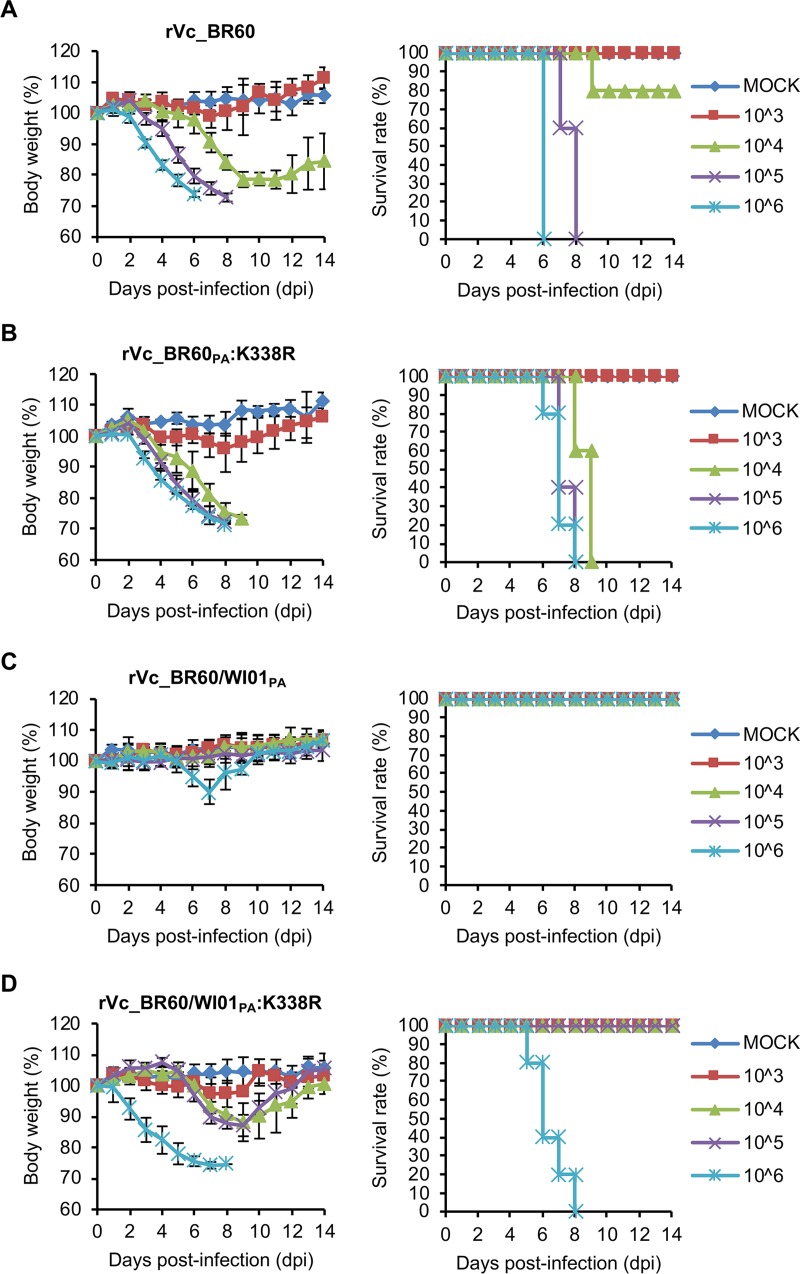
Pathogenicity of rVc_BR60 and PA K338R mutants in mice. Five mice per group were infected with 10^3^ to 10^6^ PFU of Vc_BR60 backbone recombinant viruses rVc_BR60 (A), rVc_BR60_PA_:K338R (B), rVc_BR60/WI01_PA_ (C), and rVc_BR60/WI01_PA_:K338R (D). Body weight changes and survival rates of the infected mice were monitored for 14 days. PBS was used for mock infection. Error bars for body weight changes denote SD.

**FIG 4:**
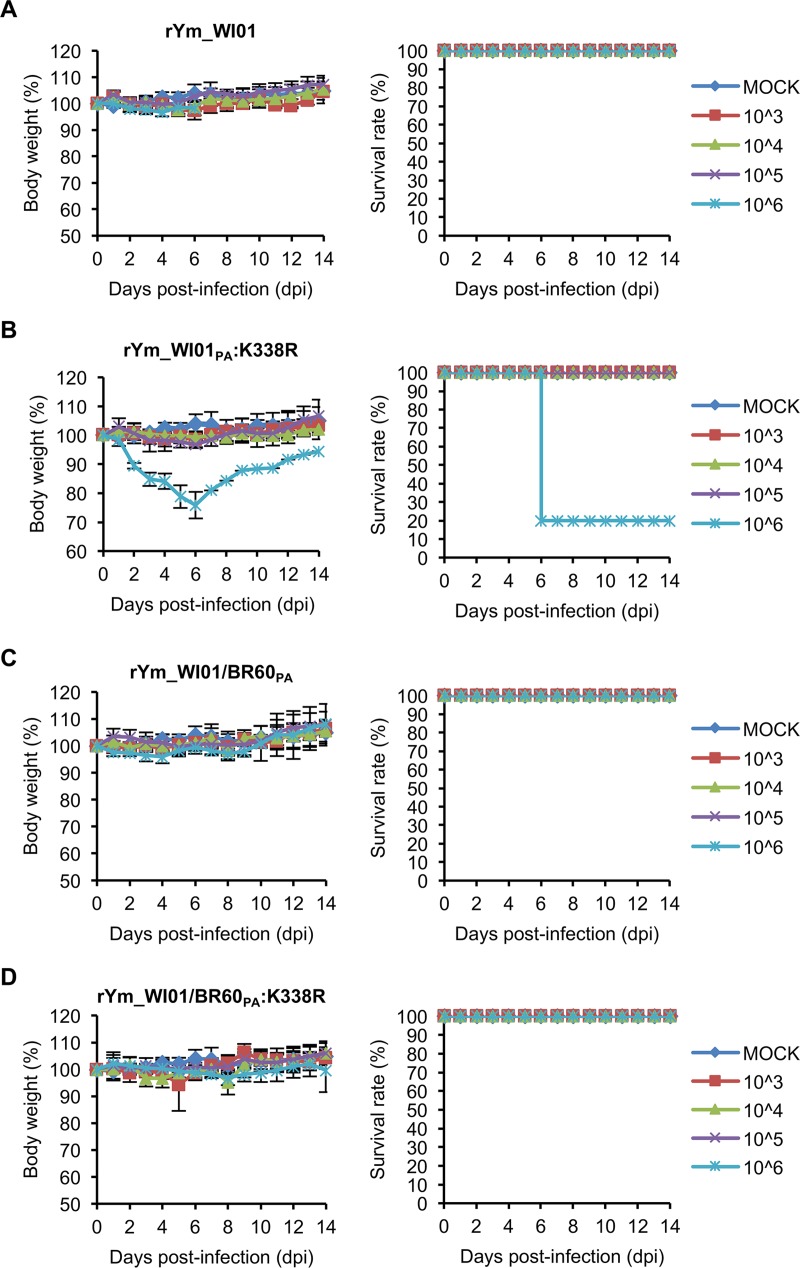
Pathogenicity of rYm_WI01 and PA K338R mutants in mice. Five mice per group were infected with 10^3^ to 10^6^ PFU of Ym_WI01 backbone recombinant viruses rYm_WI01 (A), rYm_WI01_PA_:K338R (B), rYm_WI01/BR60_PA_ (C), and rYm_WI01/BR60_PA_:K338R (D). Body weight changes and survival rates of the infected mice were monitored for 14 days. PBS was used for mock infection. Error bars for body weight changes denote SD.

To explore the effects of the PA K338R mutation on the increased pathogenicity of both mouse-adapted viruses, we infected BALB/c mice intranasally with rVc_BR60_PA_:K338R and rYm_WI01_PA_:K338R (10^3^ to 10^6^ PFU). The mice infected with rVc_BR60_PA_:K338R experienced more severe body weight losses than those infected with rVc_BR60 (Fig. 3A). The mice infected with 10^3^ PFU of rVc_BR60_PA_:K338R showed approximately 4% of maximal body weight loss at 8 dpi, and the mice infected with 10^4^, 10^5^, and 10^6^ PFU of rVc_BR60_PA_:K338R showed more than 25% body weight losses before 9, 8, and 7 dpi, respectively. The mice infected with 10^4^ PFU, 10^5^ PFU, and 10^6^ PFU of rVc_BR60_PA_:K338R were killed starting from 8, 7, and 6 dpi, respectively, and all the infected mice were dead before 10 dpi (Fig. 3B). As demonstrated with the parental Ym_WI01 virus (Fig. 1C), however, rYm_WI01 rescued through reverse genetics exhibited little pathogenicity to mice. The rYm_WI01-infected mice showed less than 5% body weight loss, and they all survived until 14 dpi (Fig. 4A). Notably, the PA K338R mutant of Ym_WI01 (rYm_WI01_PA_:K338R) killed 80% of the infected mice at 6 dpi at the highest titer of 10^6^ PFU, and the average body weight loss of these infected mice was approximately 24.9% at 5 dpi (Fig. 4B). Based on the results of body weight changes and survival rates (Fig. 3A and B and 4A and B), the MLD_50_ titers of rVc_BR60_PA_:K338R and rYm_WI01_PA_:K338R were determined to be 10^3.5^ PFU and 10^5.6^ PFU (Table 3), respectively, which were similar to those of the maVc_BR60 (10^3.5^ PFU) and maYm_WI01 (10^5.5^ PFU) viruses (Table 1). Considered together, these results indicate that the PA K338R mutation may contribute to the pathogenicity of both lineages of IBVs in mice.

**TABLE 3:** Pathological findings for the PA mutant recombinant viruses in mice

Virus	MLD_50_ (PFU)	% body wt loss (dpi)^*a*^	MDD^*b*^
rVc_BR60	10^4.4^	21.73 (5)	6
rVc_BR60_PA_:K338R	10^3.5^	24.21 (6)	7.2 ± 0.45
rVc_BR60/WI01_PA_	>10^6^	9.9 ± 3.97 (7)	ND
rVc_BR60/WI01_PA_:K338R	10^5.5^	24.97 (7)	6.4 ± 1.14
rYm_WI01	>10^6^	3.31 ± 1.52 (4)	ND
rYm_WI01_PA_:K338R	10^5.6^	20.22 ± 3.74 (5)	7.4 ± 3.71
rYm_WI01/BR60_PA_	>10^6^	4.05 ± 2.43 (4)	ND
rYm_WI01/BR60_PA_:K338R	>10^6^	3.27 ± 2.66 (8)	ND

a The mean maximum body weight loss of the mice infected with 10^6^ PFU during 14 days postinfection (dpi) is indicated with standard deviation (SD).

b The mean day of death (MDD) is indicated with SD, based on the survival rates of the mice infected with 10^6^ PFU. ND, not determined.

### Genetic compatibility for the pathogenic effects of the PA K338R mutation.

Unlike the two different phylogenetic clades of IBV HA genes (see Fig. S1A in the supplemental material), the PA genes of recent IBVs appear to belong to the same Yamagata lineage (30, 31). However, the PAs of Vc_BR60 and Ym_WI01 were distinctly located within the Yamagata lineage (Fig. S1B). Given these facts, we investigated whether the PA genes of both IBV lineage viruses could be interchangeable for the pathogenic effect of PA K338R. We generated the Vc_BR60 virus harboring the PA of Ym_WI01 (rVc_BR60/WI01_PA_), the Ym_WI01 virus harboring the PA of Vc_BR60 (rYm_WI01/BR60_PA_), and their respective PA K338R mutant viruses (rVc_BR60/WI01_PA_:K338R and rYm_WI01/BR60_PA_:K338R). In the same mouse model, the parental rVc_BR60 exhibited relatively higher pathogenicity than rYm_WI01 (Fig. 3A and 4A). However, the PA from the different lineage appeared to be less compatible for the genetic constellation of IBVs in terms of viral pathogenicity in mice. When the PA of Ym_WI01 was reassorted with rVc_BR60 (rVc_BR60/WI01_PA_), the pathogenicity of rVc_BR60 was reduced, and all the mice infected with rVc_BR60/WI01_PA_ survived until 14 dpi (Fig. 3C). Intriguingly, once again, the PA K338R mutation increased the pathogenicity of rVc_BR60/WI01_PA_. The mice infected with 10^6^ PFU of rVc_BR60/WI01_PA_:K338R experienced more than 25% body weight loss, and they all died before 9 dpi (Fig. 3D). In contrast to the results observed for the rVc_BR60/WI01_PA_ and rVc_BR60/WI01_PA_:K338R viruses (Fig. 3C and D), however, the PA K338R mutation of Vc_BR60 did not alter the pathogenicity of the rYm_WI01/BR60_PA_ virus (Fig. 4C and D). Even though the pathogenicity of rVc_BR60 was higher than that of rYm_WI01 in mice, the PA of rVc_BR60 did not change the pathogenicity of rYm_WI01 at all, and the PA K338R mutation (rYm_WI01/BR60_PA_:K338R) did not increase viral pathogenicity, either (Fig. 4C and D). These results suggest that the PA K338R mutation may be a pathogenic determinant exhibiting its effect across different IBV lineages. However, the genetic background and resultant overall genetic context are also a significant factor affecting IBV pathogenicity.

### Effects of the PA K338R mutation in ferrets.

**FIG 5:**
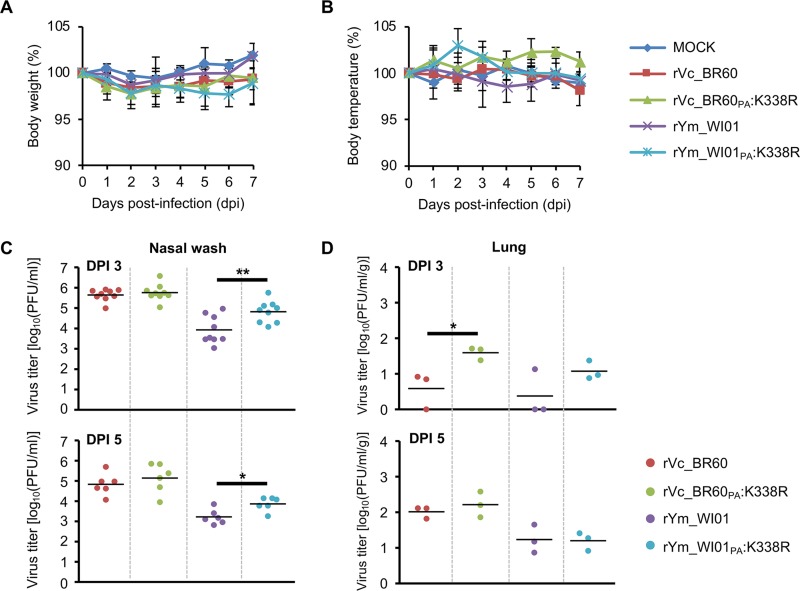
Pathogenicity of rVc_BR60, rYm_WI01, and their PA K338R mutants in ferrets. Ferrets were infected with Vc_BR60 backbone (WT and PA K338 mutant) and Ym_WI01 backbone (WT and PA K338 mutant) viruses. (A and B) Body weight (A) and temperature (B) changes were monitored for 7 days. (C and D) Viral titers in nasal wash samples (collected at 1, 3, and 5 dpi) (C) and in lungs (collected at 3 and 5 dpi) (D) were determined by plaque assay. PBS was used for mock infection. Mean values are indicated with a solid line. **, *P* < 0.01; ***, *P* < 0.001 (compared with the virus titers of rVc_BR60 and rYm_WI01, respectively).

To further evaluate the effects of the PA K338R mutation, we determined the replication titers of rVc_BR60, rVc_BR60_PA_:K338R, rYm_WI01, and rYm_WI01_PA_:K338R in the upper and lower respiratory tracts of ferrets, which are the animal model often used for studies of influenza virus pathogenicity (32, 33). With a 10^6^ PFU infection titer of each virus, ferrets exhibited less than 5% body weight losses (Fig. 5A), and their body temperatures fluctuated within an approximately 5% range (Fig. 5B). In nasal washes, rVc_BR60 backbone viruses resulted in higher viral replication than rYm_WI01 backbone viruses, and rYm_WI01_PA_:K338R exhibited statistically significantly higher replication than rYm_WI01 (at 3 dpi, *P* < 0.01; at 5 dpi, *P* < 0.05), whereas these differences were barely observed for rVc_BR60 backbone viruses (Fig. 5C). In the lungs of ferrets (Fig. 5D), the PA K338R mutants also resulted in higher replication titers than their respective parental viruses. However, due to much-reduced replication (Fig. 5D), no significant histopathologic sequelae were observed in the ferret lungs (see Fig. S2 in the supplemental material). As with the results of mouse experiments (Fig. 1 to 4), these results of ferret experiments may substantiate the effects of PA K338R on IBV pathogenicity.

### Effects of the PA K338R mutation on viral polymerase complex activity.

**FIG 6:**
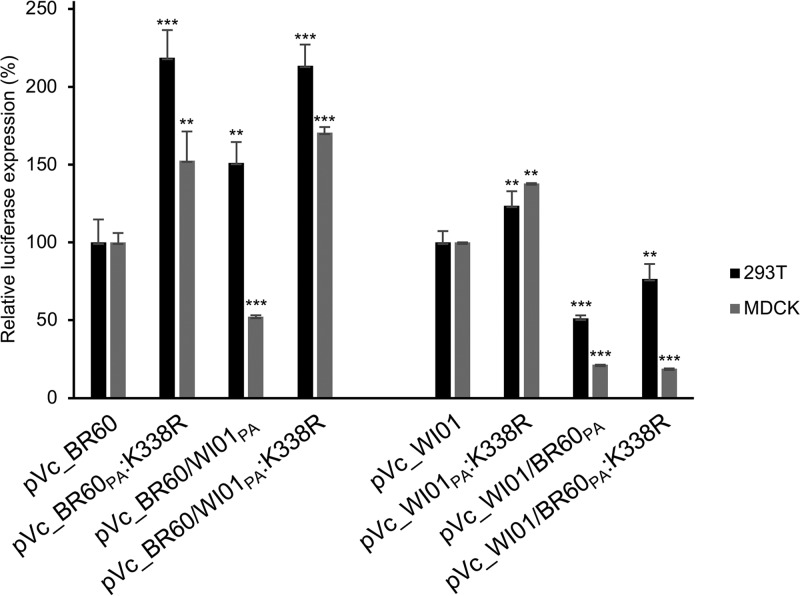
Relative activity of IBV polymerase complexes harboring WT PA and the K338R mutant. 293T and MDCK cells were transfected with the GLuc minigenome plasmid and expression plasmids of the IBV polymerase complex (PB2, PB1, PA, and NP). Luciferase expression was measured from three different experiments. The average relative results were then determined based on the luciferase expression of either WT Vc_BR60 or Ym_WI01. Error bars denote SD. **, *P* < 0.01; ***, *P* < 0.001 (compared with the expression of pVc_BR60 and pYm_WI01, respectively).

Because the PA protein consists of the viral polymerase complex of IBVs (1), we then investigated how the PA K338R mutation affects viral polymerase complex activities of IBVs. By determining luciferase expression levels in a minireplicon assay, we analyzed the activities of Vc_BR60 and Ym_WI01 backbone viral polymerase complexes. As seen in the viral pathogenicity analyses in mice, the viral polymerase complexes harboring the PA K338R mutation (pVc_BR60_PA_:K338R and pYm_WI01_PA_:K338R) enhanced luciferase expression of the respective WT polymerase complexes (pVc_BR60 and pYm_WI01) in human embryonic kidney (293T) and Madin-Darby kidney (MDCK) cells (Fig. 6). These effects of PA K338R were similarly observed in the results of polymerase complex combinations with the different PA lineages (pVc_BR60/WI01_PA_:K338R and pYm_WI01/BR60_PA_:K338R). However, as demonstrated with the reduced pathogenicity of rYm_WI01/BR60_PA_ and rYm_WI01/BR60_PA_:K338R, pYm_WI01/BR60_PA_ and pYm_WI01/BR60_PA_:K338R showed much-reduced luciferase expression compared with pYm_WI01 and pYm_WI01_PA_:K338R (Fig. 6). These results indicate that the PA K338R mutation upregulates the functional activity of the viral polymerase complex of IBVs, which may eventually affect viral pathogenicity, only within the compatible genetic background and constellation.

## DISCUSSION

Compared with IAVs, IBVs have been rarely reported in animal hosts other than human beings (34). Given the cocirculation of the two antigenically different lineages of IBVs (31, 35) and the severe morbidity of IBVs in children and adolescents (34, 36), more attention should be paid to IBV pathogenicity. However, little has been elucidated for IBV pathogenicity and its molecular determinants. In this regard, our finding, the PA K338R mutation, may have significance for assessments of health risks of IBVs.

Increased viral pathogenicity in association with increased polymerase activity has been well documented for IAVs (37–39). Our data suggest that IBV pathogenicity also could be closely related to the alteration of viral polymerase complex activity. As rVc_BR60_PA_:K338R showed higher pathogenicity than rVc_BR60 in our mouse and ferret models (Fig. 3 and 5), pVc_BR60_PA_:K338R showed higher polymerase activity than pVc_BR60 (Fig. 6). This relationship was also seen between the viral polymerase complex of Ym_WI01 virus (Fig. 6) and its pathogenicity in the tested animals (Fig. 4 and 5). Even though enhanced viral polymerase complex activities resulting from the PA K338R mutation were not sufficiently correlated with the pathogenicity of rVc_BR60/WI01_PA_:K338R and rYm_WI01/BR60_PA_:K338R (Fig. 3 and 4), the PA K338R mutation affected the viral replication competence of rVc_BR60 and rYm_WI01 in the upper and lower respiratory tracts of ferrets (Fig. 5C and D). Given all of these results, the PA K338R mutation can be suggested for its effects on IBV pathogenicity, which might result from the change of the functional activity of the viral polymerase complex (Fig. 6).

In our study, mouse lung-to-lung blind passage was performed every 2 days, so this adaptation procedure was likely to select viral clones with better fitness (22–27). In this regard, mouse-adapted viruses may result in the enhancement of replication. However, there were no significant differences in viral replication kinetics between the parental and PA K338R mutant viruses under the tested temperature and infection conditions (see Fig. S3 in the supplemental material). Given these results, it should be noted that innate immune responses (40) and various types of cells in the respiratory tracts of mice (41) might be also related to IBV replication. In our study, various viral genetic mutations were observed during adaptation in mice, so the individual and/or combined effects of these mutations on viral characteristics should be considered. As shown in Table 2, several amino acid mutations were identified from both maVc_BR60 and maYm_WI01. All of these mutations may cooperate one another to determine the overall virulence of maVc_BR60 and maYm_WI01. However, we paid attention to the PA K338R mutation because it was the only mutation identified from both maVc_BR60 and maYm_WI01, and this mutation alone appeared to transform IBV pathogenicity in mice (Fig. 4) and ferrets (Fig. 5).

Along with the PB2 and PB1 proteins, the PA protein is a member of the polymerase complex of IBVs. According to the polymerase complex structure (PDB ID 5FMZ) of IBVs proposed by Thierry et al. (42), the N-terminal region of PB2, PB1, and the C-terminal region of PA comprise the polymerase complex core (see Fig. S4 in the supplemental material), and PA residue 338 appears to be located inside this polymerase complex core, which possibly interacts with viral RNA promoter for viral transcription. In addition, PA residue 338 is found as one of the amino acid residues in α-helices of the PA protein structure, so the PA K338R mutation might eventually affect the polymerase complex activity and pathogenicity of IBVs (Fig. 3 to 6). Interestingly, in a recent study reported by Chai et al. (43), the authors adapted the Vc_BR60 strain in MDCK cells under the pressure of an HA-specific human monoclonal antibody, and they observed the PA K338R mutation along with other mutations in the PB2-, PB1-, PA-, NA-, BM2-, and NS1-coding regions. Given that the PA K338R mutation can arise during the serial adaptation of IBVs in mice (our results) or in cell cultures (43), it should be noted that, as identified in some seasonal IBV strains (see Table S1 in the supplemental material), the PA K338R mutation that exhibited increased viral pathogenicity in mice and ferrets may also arise in circulating IBVs. As presented in Table S1, we identified six strains harboring PA R338 (B/Lee/1940, B/Hong Kong/05/1972, B/Hong Kong/8/1973, B/Harbin/7/1994, B/Townsville/1/2014, and B/Hawaii/16/2015) from the Influenza Research Database (44). The PAs of these viruses appeared to be clustered into various genetic clades within the Yamagata lineage PAs (see Fig. S1B in the supplemental material). Given the reassortment incompatibility of the PA K338R mutation of Vc_BR60 with other viral polymerase complex protein genes (Fig. 6) and genetic segments of the Ym_WI01 background (Fig. 4), circulation of IBVs harboring PA R338 may be self-limited in terms of viral fitness or overall genetic context. As suggested previously (30, 31), reassortment compatibility among the viral genetic segments of the two IBV antigenic lineages may determine genetic diversity and perpetuation of IBVs in humans, which may suggest lesser effects of clonal selection on IBV evolution than on that of IAVs (2, 45). In addition, given that most of the currently circulating IBVs retain the Yamagata lineage PAs (Fig. S1B) (30, 31), it could be expected that viral pathogenicity and polymerase complex activity of rYm_WI01/BR60_PA_ and rYm_WI01/BR60_PA_:K338R might be comparable to those of rYm_WI01 and rYm_WI01_PA_:K338R. However, in contrast to our expectation, rYm_WI01/BR60_PA_ and rYm_WI01/BR60_PA_:K338R appeared to be much less fit (Fig. 4 and 6). Hence, due to the presence of various genetic clades within the PA of Yamagata lineage IBVs (31), not the genetic lineage of certain genetic segments but the overall gene constellation of IBVs should be considered for the effects of PA K338R on IBV pathogenicity.

By showing that PA K338R could render both homologous and heterologous IBV pathogenicity, we demonstrated that specific amino acid signatures at PA residue 338 might determine IBV pathogenicity. Given the appearance of the K338R mutation in the PAs of both Victoria and Yamagata lineage viruses during adaptation in mice and the increased virulence *in vitro* and *in vivo* of the PA K338R mutants, we here suggest that the PA K338R mutation may be one of the molecular determinants of IBV pathogenicity and that its potential mechanism might be closely related to the change of the biological function of the viral polymerase complex of IBVs. We believe that our results can extend our understanding of molecular interactions or reassortment compatibility between the two IBV lineages and be useful for the assessment of public health risks of IBVs.

## MATERIALS AND METHODS

### Ethics statement.

This study was conducted in strict accordance with the recommendations in the Guide for the Care and Use of Laboratory Animals of the Animal, Plant, and Fisheries Quarantine and Inspection Agency of Korea. The experimental protocols using mice, ferrets, and embryonated chicken eggs were approved by the Institutional Animal Care and Use Committee of Korea University College of Medicine (permit number KUIACUC-2014-249).

### Viruses and cells.

Two different lineages of influenza B virus, B/Brisbane/60/2008 (BR60) and B/Wisconsin/01/2010 (WI01), representing the Victoria and Yamagata lineages, respectively, were provided by the Korea Center for Disease Control (KCDC), Osong, Republic of Korea. With intranasal infection of Vc_BR60 and Ym_WI01 in BALB/c mice (female, 6 weeks old; NaraBiotech, Seoul, Republic of Korea), the first-passage viruses were recovered from mouse lung homogenates, and after the 10th serial lung-to-lung passage, maVc_BR60 and maYm_WI01 were obtained by plaque purification in MDCK cells. The viruses were propagated in 10-day-old embryonated chicken eggs (Orient Bio, Seongnam, Republic of Korea) for 48 to 72 h and confirmed by sequence analysis before use. Viral titers were determined by a hemagglutination (HA) assay using 0.5% (vol/vol) turkey red blood cells (RBC) (Innovative Research, Novi, MI, USA) and a plaque assay in MDCK cells.

MDCK and 293T cells were purchased from the American Type Culture Collection (Manassas, VA, USA). The cells were maintained in Eagle's minimum essential medium (EMEM) (for MDCK cells) (Lonza, Basel, Switzerland) and Dulbecco's modified Eagle's medium (for 293T cells) (Lonza) with supplementation of fetal bovine serum (Biotechnics Research, Lake Forest, CA, USA) and penicillin-streptomycin (Gibco, Thermo Fisher Scientific, Waltham, MA, USA).

### Animal experiments.

The BALB/c mice were used for the adaptation and pathogenicity assessment of IBVs. The MID_50_ was determined by intranasal infection with 10^1^ to 10^6^ PFU of each virus in five mice per IBV strain. The mouse lungs were harvested at 2 dpi, and the lung homogenates were used for the titration by the plaque assay. The MID_50_ titers, i.e., the lowest viral infection titer that results in the presence of infectious virus in 50% of the infected mice, were calculated using the method described by Reed and Muench (46). The MLD_50_ was determined using an infection scheme similar to that for MID_50_ determination. The infected mice were observed for 14 days for body weight changes and survival rates. Mice that lost more than 25% of their initial body weight were considered dead and were euthanized humanely. The MLD_50_ titers, i.e., the lowest viral infection titer that resulted in death of 50% of the infected mice, were also calculated using the method of Reed and Muench. For viral replication in mouse lungs and histopathology assessments, nine mice were inoculated at 10^4^ and 10^6^ PFU of Vc_BR60 and Ym_WI01, respectively, and their lungs were collected at 1, 3, and 5 dpi. For the titration of viruses, the lung samples were homogenized in EMEM with a TissueLyser II (Qiagen, Hilden, Germany). The lung samples collected at 5 dpi were prepared for hematoxylin and eosin (H&E) staining by a standard protocol and examined under a light microscope (Axioskop 40; Carl Zeiss, Oberkochen, Germany).

Ferrets (male, 18 weeks old; Wuxi Sangosho Biotechnology, Jiangsu, China) were also used for pathogenicity assessment of IBVs. Nine ferrets per group were infected intranasally with 10^6^ PFU of IBVs. The infected ferrets were observed for 7 days for body weight and body temperature changes. At 3 and 5 dpi, nasal wash samples were collected from each ferret and used for virus titration by the plaque assay. The lungs of infected ferrets were collected at 3 and 5 dpi and used for virus titration and histopathology study.

### Generation of recombinant viruses.

Vc_BR60 and Ym_WI01 backbone recombinant viruses were generated by plasmid-based reverse genetics (29). Briefly, the eight viral genetic segments were first cloned into the bidirectional pDZ vector. The plasmids were then transfected into MDCK and 293T cocultured cells using X-tremeGENE (Roche, Basel, Switzerland). The transfected cells were then maintained in EMEM and supplemented with TPCK (tosylsulfonyl phenylalanyl chloromethyl ketone)-treated trypsin at 24 h posttransfection. Viral presence was determined by the HA assay, and each virus was prepared by plaque purification and propagation in embryonated chicken eggs. Viral genetic sequences were then confirmed by reverse transcription-PCR before use.

### Polymerase activity analysis.

A minigenome plasmid was constructed by inserting the Gaussia luciferase (GLuc) reporter gene between the noncoding regions of the NA genes of both IBV lineages. The minigenome plasmid with various combinations of viral polymerase complex plasmids (PB2, PB1, and NP), including WT PA or PA K338R, were transfected into 293T or MDCK cells using X-tremeGENE (Roche). At 24 h posttransfection, luciferase expression was determined using the BioLux Gluc assay kit (New England BioLabs, Ipswich, MA, USA). Normalization was performed using WST-1 (Roche). Mean values and standard deviations (SD) of luciferase expression were determined based on those of the WT Vc_BR60 and Ym_WI01 polymerase complexes.

### Statistical analysis.

The statistical significance of virus titer differences (in the lungs of mice and ferrets and in the nasal wash samples of ferrets) and relative luciferase expression was analyzed using Student's *t* test (*, *P* < 0.05, **, *P* < 0.01; ***, *P* < 0.001).

## Supplementary Material

Supplemental material
