# Supplementary material for: A Single Amino Acid in the Polymerase Acidic Protein Determines the Pathogenicity of Influenza B Viruses
Source: J Virol. 2018 Jun 13;92(13):e00259-18. doi: 10.1128/JVI.00259-18 (PMC6002706; doi:10.1128/JVI.00259-18)
Supplement: Supplemental material [file supp_92_13_e00259-18__index.html]

A Single Amino Acid in the Polymerase Acidic Protein Determines the Pathogenicity of Influenza B Viruses — Supplemental material 

# A Single Amino Acid in the Polymerase Acidic Protein Determines the Pathogenicity of Influenza B Viruses

## Supplemental material

- Supplemental file 1 -

  Fig. S1 (Phylogenetic relationships of HA and PA genes of IBVs.)

  Fig. S2 (Effects of the PA K338R mutation on IBV pathogenicity in ferrets.)

  Fig. S3 (Growth kinetics analysis of rVc\_BR60, rYm\_WI01, and their PA K338R mutants in MDCK cells.)

  Fig. S4 (PA 338 and its neighboring residues in the crystal structure of IBV PA.)

  Table S1 (IBV strains harboring the PA K338R mutation.)

  PDF, 5.6M
