# Supplementary material for: A Single Amino Acid in the Polymerase Acidic Protein Determines the Pathogenicity of Influenza B Viruses
Source: J Virol. 2018 Jun 13;92(13):e00259-18. doi: 10.1128/JVI.00259-18 (PMC6002706; doi:10.1128/JVI.00259-18)
Supplement: Supplemental material [file JVI.00259-18_zjv013183633s1.pdf]

SUPPLEMENTAL MATERIALS

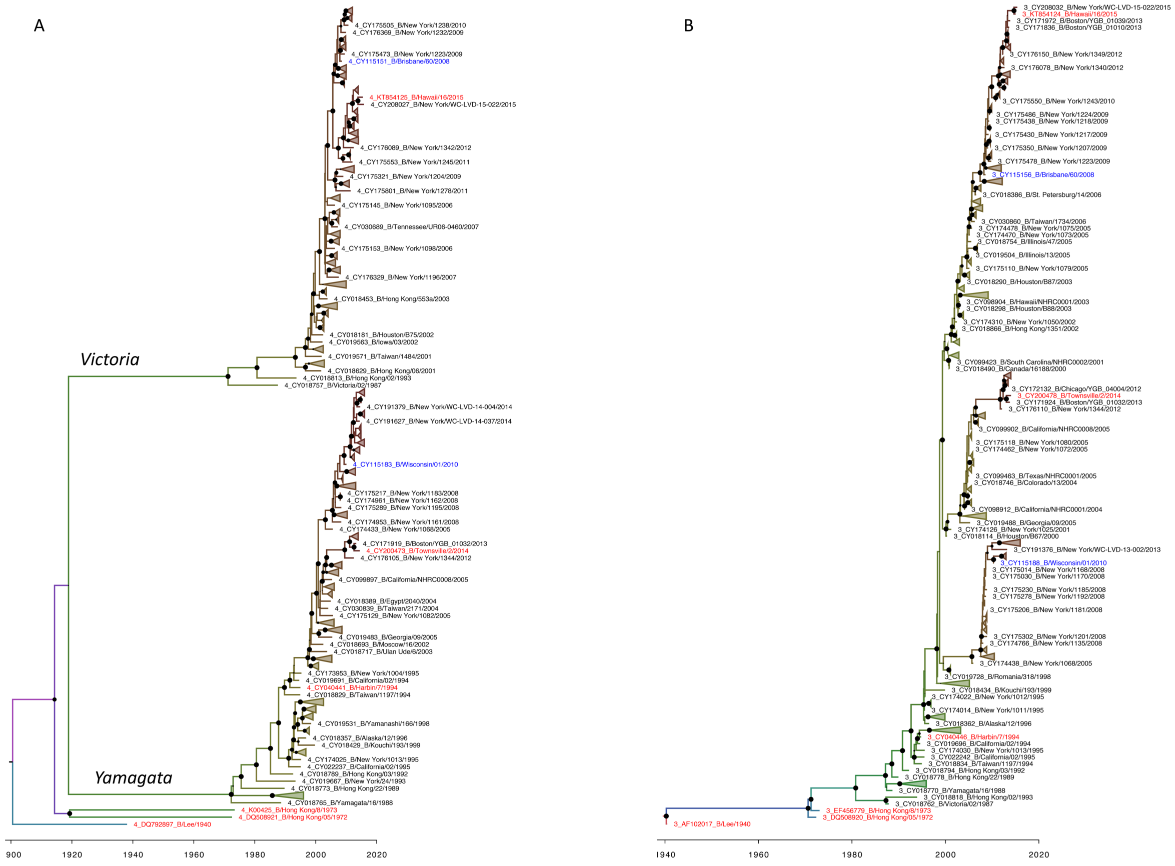

FIG S1. Phylogenetic relationships of HA and PA genes of IBVs. Phylogenetic relationships of HA (sequence number = 450) and PA (n = 450) genes of IBVs, which were downloaded from the database of Influenza Virus Resource (https://www.ncbi.nlm.nih.gov/genome/viruses/variation/flu/), were reconstructed using time-resolved Bayesian inference method implemented in BEAST (v1.8.4) (1). GTR + I + G substitution (2), uncorrelated relaxed clock with lognormal distribution, and Bayesian skygrid coalescent tree were selected for the analysis. MCMC runs were evaluated using Tracer (v1.6) (http://tree.bio.ed.ac.uk/software/tracer/). Maximum clade credibility trees were obtained using TreeAnnotator (v1.8.4) and were visualized using Fig Tree (v1.4.3) (http://tree.bio.ed.ac.uk/software/figtree/).

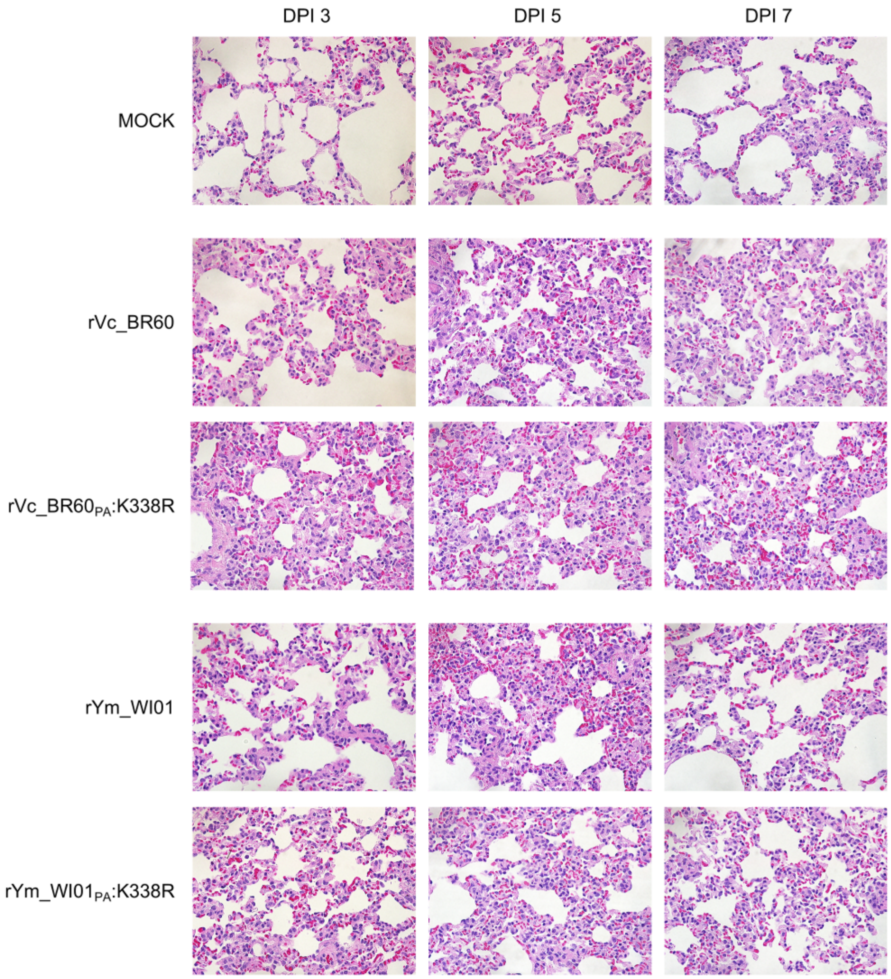

FIG S2. Effects of the PA K338R mutation on IBV pathogenicity in ferrets. The pathogenic effects of the PA K338R mutation were evaluated using H&E slides (x100 magnification) of the ferret lungs infected with Vc_BR60-backboned (WT and PA K338R mutant) and Ym_WI01-backboned (WT and PA K338R mutant) viruses. PBS was used for mock infection.

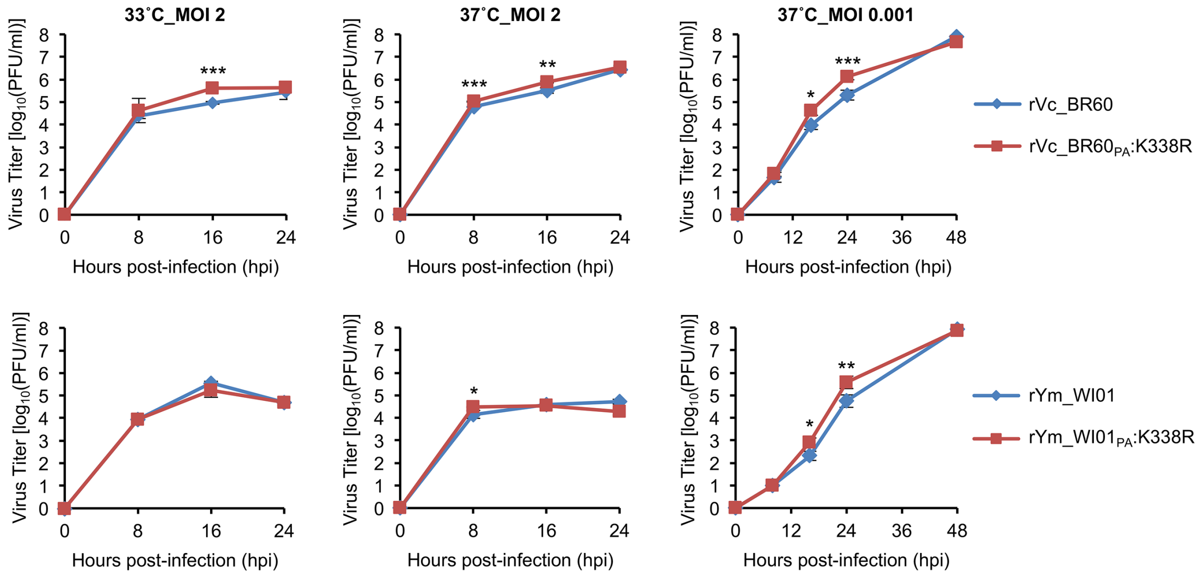

FIG S3. Growth kinetics analysis of rVc_BR60, rYm_WI01 and their PA K338R mutants in MDCK cells. Replication property of rVc_BR60, rYm_WI01 and their PA K338R mutants was evaluated in MDCK cells by changing conditions of the multiplicity of infection (MOI = 2 and 0.001) and incubation temperature (33 °C and 37 °C). For single-cycle replication condition (MOI = 2), cell supernatants were collected at 8, 16 and 24 hpi, and for multi-cycle replication condition (MOI = 0.001), cell supernatants were collected at 8, 16, 24 and 48 hpi. *, $P < 0.05$, **, $P < 0.01$ and ***, $P < 0.001$ (compared with the virus titers of rVc_BR60 and rYm_WI01, respectively).

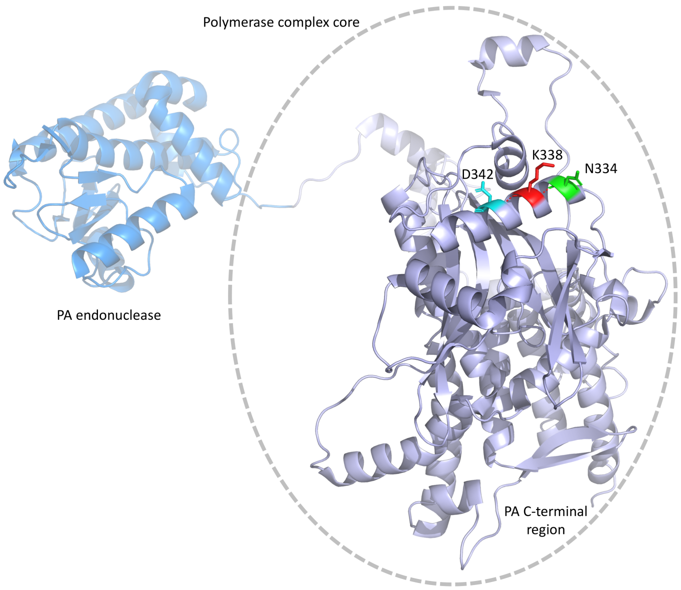

FIG S4. PA 338 and its neighboring residues in the crystal structure of IBV PA. Using the crystal structure of IBV PA protein (PDB ID: 5FMZ) (3), PA 338 and its neighboring residues were indicated with different colors (Residue 334, green; 338, red; and 342, cyan). PA endonuclease (marine blue), polymerase complex core (light blue) and C-terminal region (light blue) were also indicated based on the study of Thierry et al. (3). The PyMOL Molecular Graphics System (v2.0.6; Schrodinger LLC, https://pymol.org) was used for the visualization of PA residues and domains.

Table S1. IBV strains harboring the PA K338R mutation

Virus	GenBank aceession no.	Amino acid signature at PA residue 338
B/Brisbane/60/2008	CY115156	K
B/Wisconsin/01/2010	CY115188	K
B/Lee/1940	CY115116	R
B/Hong Kong/05/1972	DQ508920	R
B/Hong Kong/8/1973	EF456779	R
B/Harbin/7/1994	AF170570	R
B/Townsville/2/2014	CY200478	R
B/Hawaii/16/2015	KT854124	R

References

1. Drummond AJ, Suchard MA, Xie D, Rambaut A. 2012. Bayesian phylogenetics with BEAUti and the BEAST 1.7. Mol Biol Evol 29:1969-73.
2. Tavare S. 1986. Some probabilistic and statistical problems in the analysis of DNA sequences. Lectures on Mathematics in the Life Science:57-86.
3. Thierry E, Guilligay D, Kosinski J, Bock T, Gaudon S, Round A, Pflug A, Hengrung N, El Omari K, Baudin F, Hart DJ, Beck M, Cusack S. 2016. Influenza Polymerase Can Adopt an Alternative Configuration Involving a Radical Repacking of PB2 Domains. Mol Cell 61:125-37.
